# Effects of Alkalinity Stress on Amino Acid Metabolism Profiles and Oxidative-Stress-Mediated Apoptosis/Ferroptosis in Hybrid Sturgeon (*Huso dauricus* ♀ × *Acipenser schrenckii* ♂) Livers

**DOI:** 10.3390/ijms251910456

**Published:** 2024-09-27

**Authors:** Cunhua Zhai, Xiafei Liu, Yutao Li, Ruoyu Wang, Weihua Lv, Bo Ma, Dingchen Cao, Ying Zhang

**Affiliations:** 1 Key Laboratory of Cold Water Fish Germplasm Resources and Multiplication and Cultivation of Heilongjiang Province, Heilongjiang River Fishery Research Institute, Chinese Academy of Fishery Sciences, Harbin 150070, China; 2 College of Life Science and Technology, Harbin Normal University, Harbin 150025, China; 3 College of Fisheries and Life Science, Dalian Ocean University, Dalian 116023, China

**Keywords:** alkalinity stress, sturgeon, fishery breeding, ferroptosis, amino acid metabolism

## Abstract

Alkaline water is toxic to cultured aquatic animals that frequently live in pH-neutral freshwater. Overfishing and habitat destruction have contributed to the decline in the wild sturgeon population; consequently, the domestic hybrid sturgeon has become an increasingly important commercial species in China. Hybrid sturgeons are widely cultured in alkaline water, but little is known about the effects of alkalinity stress on hybrid sturgeon liver tissues. We exposed hybrid sturgeons to four alkaline concentrations (3.14 ± 0.02 mmol/L, 7.57 ± 0.08 mmol/L, 11.78 ± 0.24 mmol/L and 15.46 ± 0.48 mmol/L). Histopathology, biochemical index assessment, gene expression level detection and metabolomics analysis were used to investigate the negative effects on liver functions following exposure to NaHCO_3_. Livers exposed to alkaline stress exhibited severe tissue injury and clear apoptotic characteristics. With increased exposure concentrations, the hepatic superoxide dismutase, catalase, glutathione peroxidase and alkaline phosphatase activities significantly decreased in a dose-dependent manner. NaHCO_3_ exposure up-regulated the transcriptional levels of apoptosis/ferroptosis-related genes in livers. Similarly, the expression trends of interleukin-1β and heat shock protein genes also increased in high-alkalinity environments. However, the expression levels of complement protein 3 significantly decreased (*p* < 0.05). Hepatic untargeted metabolomics revealed the alteration conditions of various metabolites associated with the antioxidant response, the ferroptosis process and amino acid metabolism (such as beta-alanine metabolism; alanine, aspartate and glutamate metabolism; and glycine, serine and threonine metabolism). These data provided evidence that NaHCO_3_ impaired immune functions and the integrity of hybrid sturgeon liver tissues by mediating oxidative-stress-mediated apoptosis and ferroptosis. Our results shed light on the breeding welfare of domestic hybrid sturgeons and promote the economic development of fisheries in China.

## 1. Introduction

Many alkaline–saline water bodies in China have not been fully exploited because of their high salinity and pH values. The estimated area of low-lying saline–alkali water regions in China is approximately 4.6 × 10^7^ hm^2^ [1], including carbonate and bicarbonate water. The alkaline water aquaculture strategy involves the digging of ponds in areas where water is alkaline [2] and the acclimation process of animals in these ponds. However, severe injury in liver, kidney and gill structures and physiological functions can occur in animals that are reared in high-concentration alkaline water environments [1,3].

Previous studies demonstrated that alkaline stress significantly reduces the activity of antioxidant enzymes and their ability to clear excessive reactive oxygen species (ROS) in Chinese sea bass [4]. ROS can attack biomacromolecules such as proteins, lipids and nucleic acids. The enzymatic/non-enzymatic antioxidant defense systems in fish are important pathways in resisting oxidative stress injury [5]. Superoxide dismutase (SOD), catalase (CAT) and glutathione peroxidase (GSH-Px) are the main antioxidant enzymes that scavenge peroxides into water and oxygen, protecting the body from oxygen radical damage [6]. It has been shown that SOD, CAT and GSH-Px activities are changed when fish suffer from alkali stress [7,8].

When ROS production exceeds the active removal capability of organisms, oxidative stress can induce apoptosis, a programmed cell death process [9]. When cysteinyl aspartate specific proteinase-3 (Csapase3) is activated, it triggers a series of reactions that initiate irreversible apoptosis. B-cell lymphoma-2 (Bcl-2) promotes cell survival by inhibiting this apoptosis process. The Bcl-2-associated X (Bax) protein is an important pro-apoptotic factor in the Bcl-2 family [10]. Research by Tao et al. revealed that alkalinity stress can elevate the of apoptosis in *E. sinensis* by up-regulating the expression of Bax, Bcl-2 and Casepase-3 [11].

Apoptosis can suppress immune responses in fish [12,13]. When inflammation occurs in fish, interleukin-1β (IL-1β) plays an important role in mediating the inflammatory response and has been regarded as an important inflammation medium in fish [14]. Complement protein 3 (C3) is one important member in the complement system, which is responsible for natural immunity and adaptive immune regulation in vertebrate [15]. The dynamic expression mode of C3 and IL-1β may indicate the degree of immunosuppression in target organs. In Nile tilapia (*Oreochromis niloticus*), IL-1β and C3 mRNA expression after exposure to high-concentration alkalinity were significantly up-regulated and down-regulated, respectively, and the inflammatory reaction was aggravated and the immune function was inhibited upon high-alkalinity stress [16].

Ferroptosis is a programmed iron-dependent cell death form that is mediated by ROS accumulation [17]. Oxidative stress plays an important role in the ferroptosis process [18]. The cystine-glutathione (GSH)–glutathione peroxidase 4 (Gpx4) signaling pathway regulates the balance between ROS production and ferroptosis induction, while a series of ferroptosis-related factors are (in)directly regulated by Gpx4 [19]. The loss of Gpx4 activity affects the GSH synthesis process, leading to the accumulation of ROS and accelerating ferroptosis induction [20]. Cyclooxygenase 2 (COX-2) activation promotes mitochondrial ROS accumulation to mediate the oxidative stress response, which contributes to the aggregation and release of iron ions. The coding gene prostaglandin-endoperoxide synthase 2 (Ptgs2) may be a downstream regulatory molecule of ferroptosis in vitro [21].

The liver is a targeted organ in ecotoxicological studies for aquatic organisms because of its sensitivity to environmental changes [22]. Hybrid sturgeon (*Huso dauricus* ♀ × *Acipenser schrenckii* ♂) is the main species that is exploited for caviar in China; it has high economic, nutritional and scientific value. Because of its fast growth and early maturation, it is popular in the fish breeding industry [23]. It is unknown how alkalinity stress affects the hepatic metabolism functions in cultured hybrid sturgeon; therefore, we report here on the effects of alkalinity stress on the liver functions related to hepatic antioxidant capacity, tissue integrity, immune regulation and apoptosis/ferroptosis. Our results are expected to provide a scientific basis for the subsequent research and breeding of new saline–alkaline-tolerant varieties.

## 2. Results

### 2.1. Histopathology Detection

**Figure 1:**
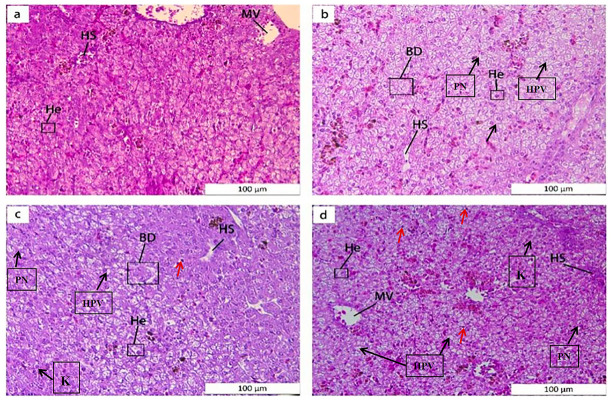
Histological observations (H&E staining, 200×) of sturgeon liver tissues exposed to different concentrations of NaHCO_3_ at 148 h: (**a**–**d**) liver tissues of C, T1, T2 and T3 groups, respectively. Note: HS, hepatic sinusoid; MV, muscular vein; HE, hepatocyte; BD, bile duct; hepatocytes hypertrophy, black arrows; HPV, hepatocellular vacuolation; PN, cellular peripheral nucleus; K, karyolysis; Pyknosis, red arrows.

Liver tissue alterations were apparent in groups T1–T3 after 148 h of NaHCO_3_ exposure (Figure 1). Hepatocytes in group C were uniformly arranged in a tight, reticulate pattern, with intact cells and centrally located nuclei. In group T1, hepatocytes were enlarged and vacuolated, and the nucleus was displaced. In groups T2 and T3, most hepatocytes were enlarged and deformed and nuclei were displaced, and more anucleate hepatocytes were apparent in group T3, accompanied by cell necrosis and lysis.

### 2.2. Apoptosis Rates Assay

**Figure 2:**
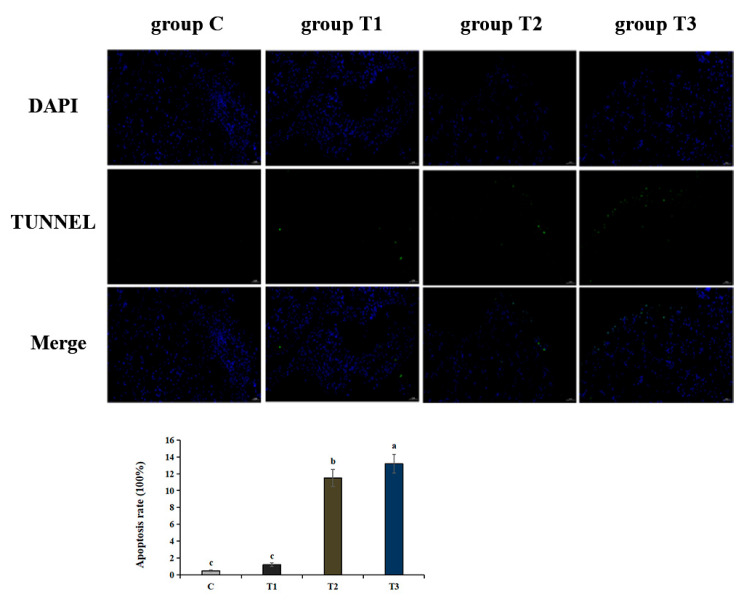
Apoptosis rates of liver cells under different alkalinity concentrations were determined using the TdT-mediated dUTP Nick-End Labeling (TUNEL) method (×200). Green fluorescence represents apoptotic cells while blue represents the nucleus. Different lowercase letters above the bars represent significant differences (*p* < 0.05) between group C, group T1, group T2 and group T3 (mean ± SD, *n* = 3).

Terminal deoxynucleotidyl transferase-mediated dUTP Nick-End Labeling (TUNEL) was used to detect apoptotic bodies. Green represented the positive apoptosis cells while blue represented the nucleus. The apoptosis ratios of group C, T1, T2 and T3 were 0.49%, 1.2%, 11.5% and 13.2%, respectively. Compared with group C, liver tissues exposed to higher NaHCO_3_ concentrations exhibited more apoptosis rates in 148 h. In addition, apoptosis was more prevalent in group T3 than in groups T1 and T2 (Figure 2), indicating that alkalinity stress would aggravate the hepatic apoptosis process in hybrid sturgeons.

### 2.3. Hepatic Oxidative Stress Indexes and Immunity Enzymes Assay

**Figure 3:**
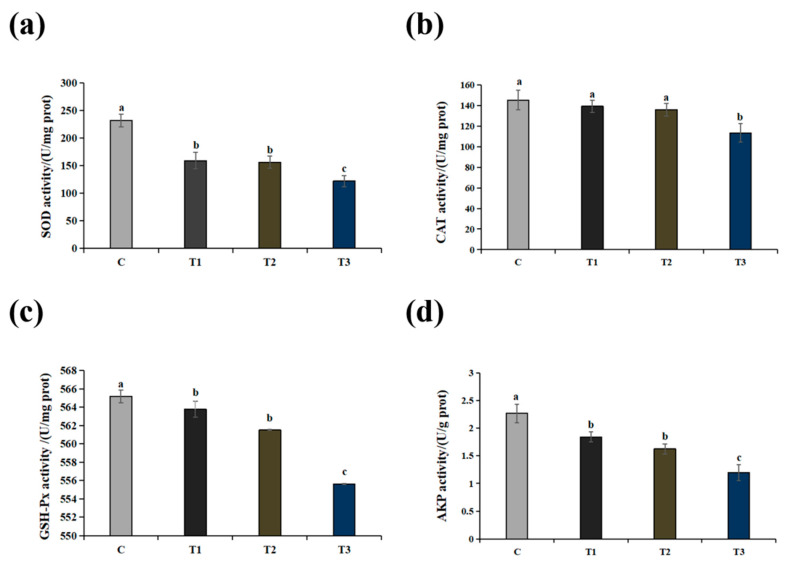
Effects of alkalinity stress on antioxidant parameters in the liver at high-alkaline exposure concentrations: (**a**–**d**) the activities of superoxide dismutase (SOD), catalase (CAT), glutathione peroxidase (GSH-Px) and alkaline phosphatase (AKP) in sturgeon liver tissues, respectively. Different lowercase letters above the bars represent significant differences (*p* < 0.05) between group C, group T1, group T2 and group T3 (mean ± SD, *n* = 3). A one-way ANOVA test was used to identify the statistical significance.

Figure 3 shows the activities of SOD, CAT, GSH-Px and AKP in livers exposed to different NaHCO_3_ concentrations. Alkalinity conditions had a significant effect on the activity of liver enzymes, and the activity of SOD, CAT, GSH-Px and AKP was significantly lower in the T3 group than in the other treatments (*p* < 0.05). Compared with the control group, the enzyme activities of hepatic SOD, CAT, GSH-Px and AKP activities decreased in all treatment groups. No significant differences were observed among T1 and T3 (*p* > 0.05).

### 2.4. Changes in Apoptosis, Immunity and Antioxidant-Related Gene Expression

**Figure 4:**
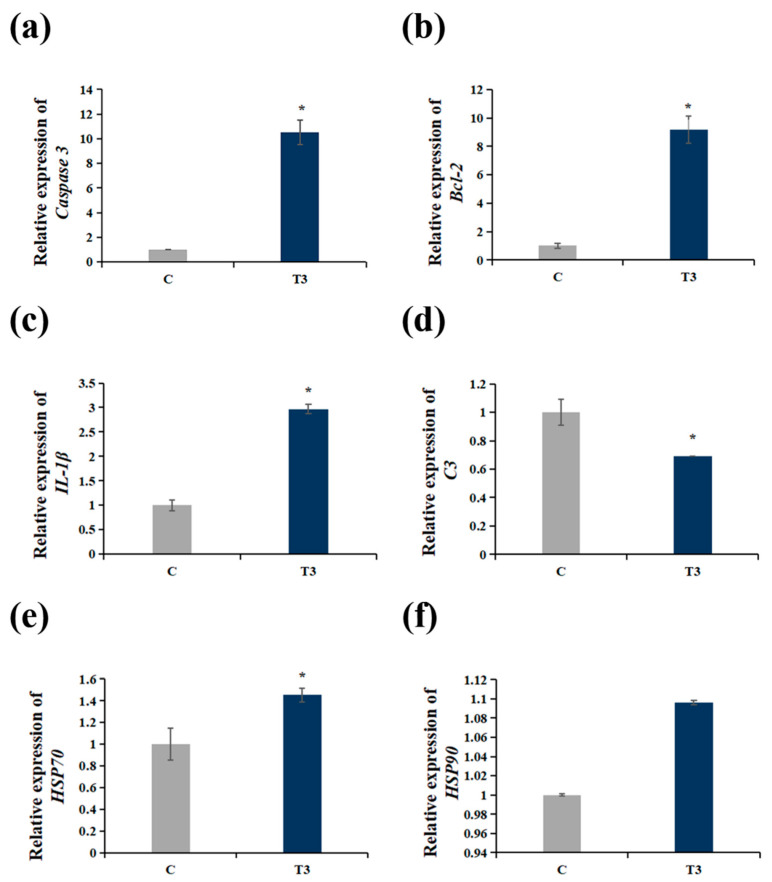
(**a**,**b**) Effects of alkalinity stress on mRNA expression levels of apoptotic-related genes (*Caspase3* and *Bcl-2*) in liver tissue. (**c**,**d**) Effects of alkalinity stress on mRNA expression levels of immune-related genes (*C3* and *IL-1β*) in liver tissue. (**e**,**f**) mRNA expression levels of stress-related gene (*HSP70* and *HSP90*). “*” above the bars represents significant differences (*p* < 0.05) between groups (mean ± SD, *n* = 9).

Figure 4 shows the effects of NaHCO_3_ exposure on the transcription levels of apoptosis, immunity and antioxidant-response-related genes at 148 h under the 15.46 ± 0.48 mmol/L NaHCO_3_ exposure condition. The relative mRNA expressions of *Caspase3*, *Bcl-2*, *IL-1β*, *HSP70* and *HSP90* increased compared with group C. Under NaHCO_3_ exposure, *Caspase3*, *Bcl-2*, *IL-1β* and *HSP70* expression in NaHCO_3_ treatments was significantly activated (*p* < 0.05) after 148 h compared with the control group. The *C3* mRNA expression levels significantly reduced in the T3 group (*p* < 0.05). There was no significant change in *HSP90* expression after the high-alkalinity treatment (*p* > 0.05).

### 2.5. Untargeted Metabolomics Analysis

**Figure 5:**
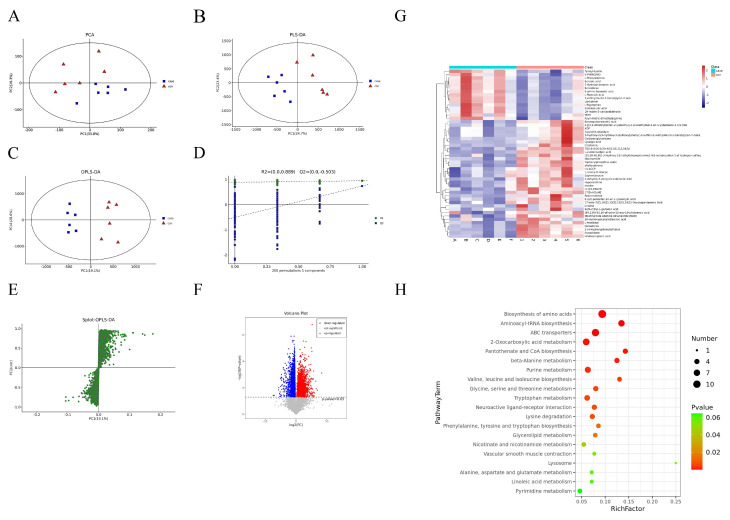
Metabolic analyses of the livers after alkalinity stress. The different metabolites between the NaHCO_3_ group (case) and the control group (con) were identified using PCA (**A**), PLS-DA (**B**), and OPLS-DA (**C**). The red triangles represent the control group. The blue square shapes represent the NaHCO3 group. (**D**) OPLS-DA permutation test. (**E**) S-Plot: the horizontal coordinate represents the effect of the metabolite on the NaHCO_3_ group and the control group, while the vertical coordinate represents the correlation between the sample and the metabolite. (**F**) The volcano plot of differential metabolites in livers. Red represents up-regulated, blue represents down-regulated and brown represents not significant. (**G**) Heatmap of differential metabolites in livers. (**H**) Scatter plot of significantly enriched KEGG pathways. The size of nodes indicates the gene number that mapped to the KEGG pathway; 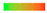 represents the *p*-value decreasing from left to right. The Y-coordinate represents the pathway term. The X-coordinate represents the relative rich factors of each pathway.

The PCA (Figure 5A), (O)PLS-DA (Figure 5B,C) and OPLS-DA permutation test (Figure 5D) results indicated that the expression levels of some metabolites differed significantly between groups C and T3 (*p* < 0.05). In the S-Plot, metabolites farther from the origin differed most (Figure 5E). In total, 325 differentially expressed metabolites (DEMs) were identified (Figure 5F), with the largest categories being “Amino acids, peptides and analogues” (12.77%), “Carbohydrates and carbohydrate conjugates” (7.17%) and “Benzenetriols and derivatives” (2.80%). Compared with group C, tyrosyl-lysine, L-phenylalanine and L-tryptophan were significantly increased, while creatine, 1,4-β-D-glucan and oxidized glutathione (GSSG) were significantly decreased (*p* < 0.05) (Figure 5G). DEMs can be enriched in a series of metabolism pathways associated with the amino acid metabolism process, including the biosynthesis of amino acids; beta-alanine metabolism; alanine, aspartate and glutamate metabolism; glycine, serine and threonine metabolism; tryptophan metabolism; valine, leucine and isoleucine biosynthesis; lysine degradation; and phenylalanine, tyrosine and tryptophan biosynthesis (Figure 5H). A Kyoto Encyclopedia of Genes and Genomes (KEGG) enrichment analysis also identified the “ferroptosis” pathway to be significantly enriched.

### 2.6. Expression Levels of Ferroptosis/Apoptosis-Related Proteins

**Figure 6:**
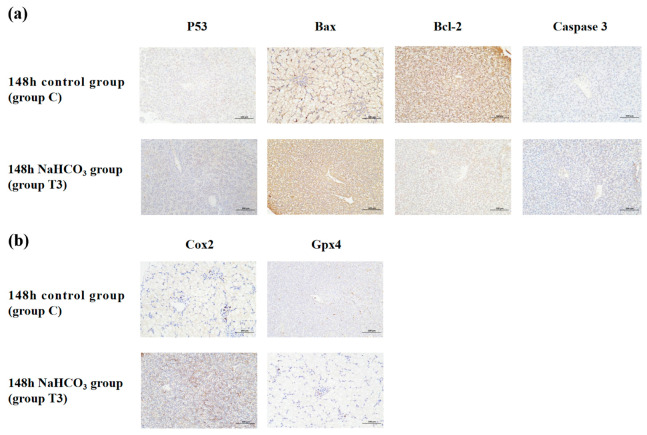
Immunohistochemical staining for (**a**) apoptosis- and (**b**) ferroptosis-related proteins in sturgeon liver tissues (400×; scale: 100 μm). The nucleus is blue, and the positive expression area of the target protein is brown.

Immunohistochemistry was used to investigate the protein expression levels of the apoptosis biomarkers P53, caspase-3, Bax and Bcl-2 and the ferroptosis regulators COX-2 and Gpx-4 (Figure 6). Compared with group C, significantly higher levels of P53, Bax, caspase-3 and COX-2 were found in group T3 (*p* < 0.05), while Bcl-2 and Gpx4 protein expression was inhibited markedly (*p* < 0.05).

## 3. Discussion

The use of alkaline water in fishery aquaculture poses a serious threat to the health of cultured aquatic species [24]. Alkaline stress can disrupt immune and antioxidant systems, weakening antioxidant enzyme activities and their ability to clear accumulated ROS [4]. Despite their tolerance to alkaline stress, the mechanism of resistance in hybrid sturgeons to alkaline culture remains unknown. In this study, we examined liver tissues of hybrid sturgeons because they are involved in metabolism, immunity and detoxification processes, and the liver is more sensitive to environmental xenobiotics.

The organisms were able to rapidly synthesize HSP family proteins when exposed to exogenous stimuli or environmental stressors. Heat shock protein 70 (HSP70) is significantly activated under physiologic stress to prevent vital protein damage and facilitate the injured cell clearance process by regulating the inflammation response [25]. A high level of HSP90 promotes self-protective regulation and helps the body adapt to environmental stress. Data in one study suggested that HSP70 and HSP90 were up-regulated after exposure to alkalinity in *Luciobarbus capito* [26]. Similarly, in this study, we found a significant increase in *HSP70* and *HSP90* gene expression levels in group T3 livers, which may be due to the stress caused by alkalinity in hybrid sturgeons. CAT and SOD represent the first line of defense to alleviate ROS toxicity [27]. GSH-Px plays an important role in neutralizing ROS and protecting the immune system [28]. When exposed to an exogenous environmental stress source, more ROS were produced to attack the mitochondrial membrane, activating the apoptosis program and initiating a compensatory response reaction to repair tissue injury. SOD transforms O^2−^ into O^2^ and H_2_O_2_, while CAT converts H_2_O_2_ to O_2_ and H_2_O; meanwhile, GSH-Px detoxifies H_2_O_2_ and organic peroxides by converting reduced GSH to GSSG [29]. Environmental alkalinity could cause oxidative stress by inhibiting SOD and CAT activities [30]. We reported the inhibition of hepatic SOD and CAT activities with the increasing NaHCO_3_ concentrations. Wei et al. [31] demonstrated that NaHCO_3_ stress could activate GSH-Px activity in the liver of crucian carp (*Carassius carassius*), but we found that GSH-Px activity would decrease with the increased trends of alkalinity concentrations at 148 h. A decline in trends in SOD, CAT and GSH-Px activities might represent the over-production of superoxide anion radicals, which leads to imbalance in the oxidative/anti-oxidative system, mediating the oxidative stress response in hybrid sturgeons under alkali stress.

We found that hepatocyte integrity is injured in hybrid sturgeon livers under the high concentration of alkalinity exposure condition, and the ratio of apoptotic cells exposed to NaHCO_3_ increased with elevated exposure concentrations, indicating that alkalinity exposure promoted apoptosis in a dose-dependent manner. We hypothesized that hybrid sturgeons exposed to alkaline conditions required more energy to maintain osmotic balance. As a consequence, the liver consumed a mass of hepatic glycogen to cause the hepatic nutrition loss, accelerating cell apoptosis and inducing structural abnormality. Our results were also consistent with those in *Eriocheir sinensis* and *Litopenaeus vannamei* [32,33]. P53 can indirectly promote Bax expression by inhibiting Bcl-2 gene expression, leading to apoptosis [34]. Under the stimulation of various apoptosis factors, mitochondria released cytochrome C (Cyt c) into the cytoplasm, which binds to apoptosis protease activating factor 1 (APAF1) and caspase-9 to form the apoptotic complex. This complex activates the downstream apoptosis executor Caspase-3, which ultimately induces the apoptosis process [35]. We also found that the mRNA of apoptosis-related genes (*Caspase-3* and *Bcl-2*) in livers significantly increased following a high concentration of NaHCO_3_ exposure. In our study, the translation of the anti-apoptotic protein Bcl-2 was inhibited and expressions of pro-apoptotic proteins P53, caspase-3 and Bax were increased, which could confirm that alkalinity exposure promoted the cell apoptosis process in the hybrid sturgeon hepatic system through the post-transcriptional modification of the *Bcl-2* gene.

Salinity and heavy metal stress can change amino acid levels in oyster (*Crassostrea virginica*) gills [36]. Amino acids could represent an important substrate for energy production in fish. Under environmental stress, amino acids could be consumed as a direct energy source to maintain physiological functions [37]. Therefore, the changes in amino acid metabolism suggested that alkalinity stress could influence the energy supply process because of increased metabolism requirements during prolonged alkalinity stress. This is supported by the decrease in the levels of a series of amino acid-dependent nutrients such as creatine, which was probably used for energy supply. In addition, existing literature has illustrated that specific amino acids are involved in the induction process of oxidative stress injury [38] by generating excessive ROS through the physiological metabolism process. For instance, endogenous phenylalanine secretion could mediate the natural antioxidant defense line and activate cascaded oxidative stress injury [39]. We identified GSSG in livers exposed to high concentrations of NaHCO_3_ stress based on the metabolomics results. We noted that oxidized glutathione played an important role in the glutathione antioxidant system, which is the primary enzymatic antioxidant defense system in fish. *Gpx4* can reduce H_2_O_2_ and lipid hydroperoxide levels by converting GSH to GSSG, thereby suppressing ROS production. ROS accumulation is an important induction factor in the ferroptosis process [19]. We reported that GSH-Px activities, *Gpx4* mRNA levels and GSSG content were down-regulated under alkali stress, while protein levels of the ferroptosis-related gene *Cox2* were significantly increased and *Gpx4* was inhibited. This indicated that the GSH-Gpx4 pathway was imbalanced, which led to ROS accumulation and oxidative stress response and thereby induced ferroptosis. Lu et al. [20] reported empagliflozin to significantly enhance *Gpx4* mRNA expression, eliminate excessive ROS accumulation and restore iron homeostasis, alleviating cellular ferroptosis in renal tubules of diabetic mice. However, typical ferroptosis characteristics should be observed in further studies.

Environmental pollution can affect the immune ability of fish. For example, in yellow catfish (*Pelteobagrus fulvidraco*) exposed to ammonia, a series of physiological and immune factors (AKP, *C3* and *IL-1β*) were altered to initiate the inflammation-development process to repair injured tissues [40]. The immune status of the organisms was closely associated with an inflammation response that was mediated by a series of molecular media. Phosphatase, a phosphate monoester hydrolase, catalyzes the hydrolysis of various phosphorus compounds and is important for detoxification in teleosts [41]. AKP is a critical alkalinity tolerance factor that regulates liver metabolism and immune response [42]. We report AKP activity in hybrid sturgeon livers to significantly decrease with NaHCO_3_ concentration at 148 h, suggesting that this non-specific immunomodulatory enzyme had reached a threshold in defense capability. Consistent with previous results, carbonate alkalinity affected AKP activity in naked carp (*Gymnocypris przewalskii*) [43]. Complement 3 (C3) is an important protein in the complement cascade and participates in complement activation and immune defense. *C3* can regulate the inflammatory response by inhibiting the expression of pro-inflammatory cytokines (*IL-1β*) and promoting anti-inflammatory cytokine synthesis (*IL-10* and *TGF-β*), alleviating tissue lesions caused by environmental stress [44]. In accord with the above studies, our results indicated that there was a general decrease in *C3* expression levels in hybrid sturgeons under high concentrations of alkalinity stress, which is similar to the results of *Oreochromis niloticus* under ammonia stress [45]. Additionally, we also found that transcription levels of *IL-1β* increased in the T3 group, indicating that the inflammation response was initiated by alkaline stress. Obvious pathological characteristics such as cell nucleus loss/displacement and hepatocyte vacuolization/lysis occurred with alkaline stress, especially in the highest concentration. These results confirmed that high-concentration alkalinity stress induced hepatic immune imbalance effects in hybrid sturgeons, accelerating the inflammatory response and severe liver injury.

## 4. Materials and Methods

### 4.1. Animal and Experimental Procedures

Our experiments comply with animal care laws and guidelines (Directive 2010(63)EU); all procedures were approved by the Laboratory Animal Ethics Committee of the Heilongjiang River Fisheries Research Institute (No. 20230925-001).

Thirty-day-old hybrid sturgeons were provided by the Hulan Farming Base, Chinese Academy of Aquatic Sciences (Harbin, China). Fish were first acclimatized in tanks for two weeks before the formal experiment. The experimental vessel was a 150.7 L indoor temperature-controlled circulating water glass tank (80.5 cm × 48 cm × 39 cm), and the water velocity was 0.18 m/s. The water was aerated tap water and changed daily. Water quality parameters were measured every six hours, in which temperature, pH and dissolved oxygen were measured using a Water Quality Meter (Thermo, 4-Star, Waltham, MA, USA) and alkalinity concentration was determined by methyl orange hydrochloride titration. The water quality indexes were maintained as follows: temperature at 15.40 ± 0.6 °C, alkalinity at 3.14 ± 0.02 mmol/L, dissolved oxygen > 6 mg/L, and a pH range of 6.7 ± 0.12. During the adaptation period, the fish were fed with commercial feed. All the fish were kept in 12 h of light/12 h of darkness.

One hundred twenty healthy fish (body length 133.17 ± 16.75 mm; body weight 12.43 ± 3.99 g) were selected and randomly allocated to twelve tanks (4 treatments, three tanks per treatment, ten fish per tank). Control treatment (C) tanks were filled with tap water; test treatment (T) tanks were filled with fresh tap water that contained dissolved 99% pure NaHCO_3_. Alkalinity concentration was determined by methyl orange hydrochloride titration. For detailed procedures, please refer to the previous literature [46].

Based on water quality standards for aquaculture in saline–alkaline land (GB/T 43563-2023 [47]), the salinity threshold value in water bodies for sturgeon culture is lower than 10.0 mmol/L. Therefore, NaHCO_3_ concentrations in the current study were set as follows: 3.14 ± 0.02 mmol/L (C), 7.57 ± 0.08 mmol/L (T1), 11.78 ± 0.24 mmol/L (T2) and 15.46 ± 0.48 mmol/L (T3). Fish were fed twice daily with commercial feed (Shengsuo, Yantai, China) at 1% of their body weight daily; feed nutrient contents are presented in Table S1. After feeding for 30 min, residual feed and fecal material were removed from each tank; 30% of the water in tanks was replaced daily with fresh water with the same NaHCO_3_ concentration. No dead individuals were observed during the experiment period. The physical and chemical parameters in the water were monitored during the formal experiment period: dissolved oxygen was >9 mg/L, the water temperature ranged from 16 to 17 °C and the light cycle was 12 h light/12 h dark.

### 4.2. Sampling

Nine fish (three fish per tank) in each group were randomly selected from each treatment group after 148 h. Fish were immediately anesthetized using 0.02% Tricaine methanesulfonate (MS-222; Sigma, Cream Ridge, NJ, USA). Body length and weight were determined, and liver tissues were excised and divided into three portions: one for RNA extraction and untargeted metabolomics analysis (frozen in liquid nitrogen and then stored at −80 °C), one for histopathology, immunohistochemistry and apoptosis detection (fixed in 10% formaldehyde), and one for enzyme activity assessment.

### 4.3. Histopathology Detection

After fixation for 48 h, tissue samples were dehydrated through a graded series of ethanol, followed by clearing in xylene, embedding in paraffin blocks and then sectioning (5–6 μm thickness) using an HM 325 microtome (MICROM, München, Germany). Sections were stained with hematoxylin and eosin (HE). Slides were observed under a light microscope (Eclipse Ci-L, Nikon, Tokyo, Japan) and photographed (×200).

### 4.4. Apoptosis Rates Assay

After paraffin-embedded liver tissues were sectioned, slides were dewaxed with different concentrations of ethanol (100%, 95%, 90%, 80% and 70%) and xylene at room temperature. After gradient dewaxing, sections were immersed in a 1% phosphate-buffered saline (PBS) solution, proteinase K was added to the sections, and they were incubated at room temperature for 25 min. DNAzyme was then added, and the sections were incubated at room temperature for 7 min. Residual buffer was discarded, and this step was repeated three times followed by the addition of 5× Equilibration Buffer and incubation for 25 min. The slides were then incubated with terminal deoxynucleotidyl transferase (TdT) at 37 °C in the dark, counterstained with 4′,6-diamidino-2-phenylindole (DAPI), dehydrated with different gradients of alcohol, and de-etherified with xylene. The slides were observed under a microscope (Eclipse Ci-L, Nikon, Japan) and photographed three times per sample within 10 min to avoid fluorescence quenching.

### 4.5. Measurement of Physiological Indexes

SOD, CAT, GSH-Px, AKP and total protein (TP) were detected using kits purchased from the Nanjing Jiancheng Bioengineering Institute (Nanjing, China). Liver samples were homogenized and diluted with cold physiological saline at a ratio of 1:9 (*w*:*v*). After centrifugation at 3500 rpm for 10 min at 4 °C, the supernatant was collected. Enzyme activities were measured following the kit manufacturer’s instructions. Optical density (OD) values were determined using the absorbance microplate reader (SpectraMax Plus 384, Molecular Devices, San Jose, CA, USA) at 550 nm (SOD), 405 nm (CAT), 412 nm (GSH-Px), 520 nm (AKP) and 562 nm (TP), respectively. Based on the TP concentrations in each sample, the contents for each physiological indicator were calculated according to the corresponding formulas. TP concentrations were measured using the Coomassie brilliant blue G-250 method.

### 4.6. Relative mRNA Expression Detection

Primers were designed using the Oligo 6.0 online program based on apoptosis, immunity and HSP family-related gene sequences that were published on GenBank. All primers were synthesized by Kumei Biotechnology Co., Ltd. (Changchun, China) (Table S2). We verified the feasibility of the selected genes and primers before performing a real-time quantitative polymerase chain reaction (qRT-PCR). Total RNA was isolated from liver tissues using the TRIzol reagent (Invitrogen, Carlsbad, CA, USA) following the manufacturer’s protocols. RNA concentration and quantity were determined using a NanoDrop ND-1000 spectrophotometer (Thermo Fisher Scientific, Wilmington, DE, USA), and RNA integrity was evaluated by electrophoresis using 1% agarose gels. The first-strand cDNA was synthesized from 1 μg of total RNA using a Prime Script^TM^ RT reagent kit with DNA Eraser (TaKaRa, Osaka, Japan). Then, a total of 9 cDNA samples were diluted 10-fold with nuclease-free water and utilized as templates. qRT-PCR was performed in the ABI 7500 real-time PCR instrument (Thermo Fisher Scientific). Each 10 μL qRT-PCR reaction contained 5 μL 2×TB Green Premix Ex Taq II (Tli RNaseH Plus, TaKaRa, Osaka, Japan), 0.4 μL of each forward and reverse primer, 3 μL sterile distilled H_2_O (dH_2_O), 1 μL template cDNA and 0.2 μL 50×ROX Reference Dye II (Roche, Switzerland). Detailed cycle parameters were set at 95 °C for 180 s, 40 cycles of 95 °C for 5 s, 60 °C for 15 s and 72 °C for 30 s. The 2^−ΔΔCT^ method was used to calculate relative gene expression levels using *β-actin* (Forward, 5′-ATCGCCGCACTGGTTGTTGA-3′; Reverse, 5′-ATGCCGTGCTCGATGGGATA-3′) and the gene was chosen as the reference gene [48].

### 4.7. Metabolomics Analysis

Untargeted metabolomics was performed to identify differential metabolites between treatments C and T3 at 148 h (six individuals per treatment). Liver samples (30 mg) were transferred to sterile Eppendorf tubes, and 20 µL of an internal standard (0.3 mg/mL 2-chloro-l-phenylalanine) and 400 µL of an extraction solvent with methanol/water were added to each. Samples were stored at −80 °C for 2 min, ground at 60 Hz for 2 min, ultrasonicated at an ambient temperature (25–28 °C) for 10 min, then stored at −20 °C for 30 min. The extract was centrifuged at 13,000 rpm at 4 °C for 10 min. We added a 300 μL aliquot of the supernatant and then dried it in a freeze-centrifugal dryer. The samples were centrifuged at 13,000 rpm at 4 °C for 10 min. The supernatant was collected by a crystal syringe, filtered through a 0.22 µm microfilter and transferred to vials for further analysis. An ExionLC AC (AB SCIEX, Framingham, MA, USA) coupled with a Triple TOF 6600 plus (AB SCIEX, USA) was used to analyze hepatic metabolism profiles. An ACQUITY UPLC HSS T3 column (1.8 µm, 2.1 × 100 mm) was used in both positive and negative modes. Data acquisition was performed in full scan mode (*m*/*z* ranges from 70 to 1000) combined with IDA mode. Acquired liquid chromatograph mass spectrometer (LC-MS) raw data were analyzed using Progenesis QI software version 2.0 (Waters, Milford, MA, USA). A principle component analysis (PCA) and (orthogonal) partial least-squares-discriminant analysis (O)PLS-DA was performed on metabolomics data using the R language ropls package version 4.2.2. The (O)PLS-DA model was used to determine metabolomic data similarities between group C and T3, the variable importance in projection (VIP) value (>1) and a *p*-value < 0.05. All raw data were analyzed by Shanghai Lu-Ming Biotech Co., Ltd. (Shanghai, China).

### 4.8. Immunohistochemistry Assay

Fixed tissue was cut into sections and placed into xylene to remove paraffin then into 100% ethanol. The sections were placed in citrate buffer (pH 6.0) at 28 °C for antigen retrieval, incubated with 3% H_2_O_2_ for 25 min to inactivate endogenous peroxidase, and washed with PBS (pH 7.4) three times. After the addition of 3% bovine serum albumin (BSA), sections were sealed in a drying oven. Primary antibodies were added into sections for incubated at 4 °C overnight. The following day, biotin-labeled secondary antibody and horseradish peroxidase (HRP)-conjugated IgG were added, then incubated at 28 °C for 75 min. Fresh diaminobenzidene (DAB) color-developing solution was added and then the sections were counterstained with hematoxylin, dehydrated and mounted. Sections were scanned by CaseViewer (3DHISTECH, Budapest, Hungary); the nucleus was blue, and the positive expression area of the target protein was brown.

### 4.9. Statistical Analysis

Statistical analyses were performed using SPSS 26.0 software (IBM Corp, Armonk, NY, USA). Data normality and homogeneity of variance were first determined using Kolmogorov–Smirnov and Levene’s tests, respectively, with *p* < 0.05 considered significant. To determine if NaHCO_3_ concentration affected physiological parameter values, the normality and homogeneity of variance for physiological parameter values were first determined using Kolmogorov–Smirnov and Levene’s test, respectively, and one-way analysis of variance was used to determine significant differences among treatments. Plots were visualized using GraphPad Software (Version 8.4.3, USA); data results are described as mean ± standard deviation (mean ± SD).

## 5. Conclusions

Alkalinity stress induces oxidative stress injury in sturgeons, leading to hepatocyte apoptosis and ferroptosis. This disrupts the normal liver immune response capacity and causes severe liver damage. Additionally, high alkalinity exposure can change hepatic amino acid metabolism profiles, initiating compensatory tissue injury repair mechanisms to reduce the negative effects of alkaline exposure. We report baseline data on the effects of alkaline water aquaculture on hybrid sturgeon breeding. While alarming, further investigation into how these effects may affect hybrid sturgeon survivorship, fecundity and caviar product quality is required.

## Data Availability

The data that support the findings of this study are available from the corresponding author upon reasonable request.

## Supplementary Materials

The supporting information can be downloaded at https://www.mdpi.com/article/10.3390/ijms251910456/s1.

## Abbreviations

ROS	reactive oxygen species
SOD	superoxide dismutase
CAT	catalase
GSH-Px	glutathione peroxidase
Csapase3	cysteinyl aspartate specific proteinase-3
Bcl-2	b-cell lymphoma-2
Bax	Bcl-2-associated X
P53	tumor protein p53
PUMA	p53 up-regulated modulator of apoptosis
IL-1β	interleukin-1β
C3	complement protein 3
AKP	alkaline phosphatase
HSP70	heat shock protein 70
HSP90	heat shock protein 90
GSH-Gpx4	cystine-glutathione–glutathione peroxidase 4
COX-2	cyclooxygenase 2
Ptgs2	prostaglandin-endoperoxide synthase 2
GSSG	oxidized glutathione
