# Supplementary material for: Effects of Alkalinity Stress on Amino Acid Metabolism Profiles and Oxidative-Stress-Mediated Apoptosis/Ferroptosis in Hybrid Sturgeon (Huso dauricus ♀ × Acipenser schrenckii ♂) Livers
Source: Int J Mol Sci. 2024 Sep 27;25(19):10456. doi: 10.3390/ijms251910456 (PMC11476414; doi:10.3390/ijms251910456)
Supplement: Supplementary file 1 [file ijms-25-10456-s001.zip › ijms-3221779-supplementary.pdf]

Table S1 The nutrient content of feed for hybrid sturgeon

Nutrient content indicators (g/100g)	
Crude protein	44.1
Crude ether extract	10.3
Crude ash	18.6
Crude fiber	5.8
Ca	4.9
Total Phosphorus	1.6
Lysine	2.3
Water	12.4

Table S2 primer sequence of qPCR

Gene	Primer sequence	Product Length (bp)	Annealing temperature (°C)	Gen Bank
HSP70	F:CAACGACCAGGGTAACAGGA	171	57.3	MN636875
	R:ATATCGGCTTGCACAACACC			
HSP90	F:GACCATTGTAGACACTGGGATC	174	57.8	KM103517
	R:TTCTCAGCCACCAGGTAAGC			
Caspase3	F:GCTCTATGGAACGGACGATT	231	56.9	NM131877
	R:TAGTAGCCTGGAGCAGTGGA			
Bcl-2	F:ATGGCAAATAACACGAGACC	218	56.0	NM001030253
	R:TTTACAAAAGGGGAAGGACC			
IL-1β	F:ACCCCTTCTTTTCCTTCGTT	113	57.8	MN923070
	R:GCAAGTCATTGTCGTCGTAA			
C3	F:TGACATTGCTTGTGAACCTG	132	58.8	NM001040469
	R:GGATTTTCATCACTCCCTTC			
